# Meta-analysis of efficacy and safety of Yttrium-90 radioembolization (TARE) in the treatment of advanced hepatocellular carcinoma

**DOI:** 10.3389/fnume.2026.1784215

**Published:** 2026-03-19

**Authors:** Hongyin Lin, Quanhui Tan

**Affiliations:** 1 Department of Nursing, Shanghai Sixth People's Hospital Affiliated to Shanghai Jiao Tong University School of Medicine, Shanghai, China; 2 Department of Infectious Diseases, Shanghai Sixth People's Hospital Affiliated to Shanghai Jiao Tong University School of Medicine, Shanghai, China

**Keywords:** advanced HCC, hepatocellular carcinoma (HCC), locoregional therapy, meta-analysis, safety, survival, TARE, Yttrium-90

## Abstract

**Background:**

Transarterial radioembolization (TARE) using Yttrium-90 microspheres has emerged as a promising locoregional therapy for patients with advanced hepatocellular carcinoma (HCC). However, the efficacy and safety of TARE compared to conventional treatments remain uncertain. This meta-analysis aimed to comprehensively evaluate the survival outcomes and safety profile of TARE in advanced HCC.

**Methods:**

A systematic literature search was conducted in PubMed, Embase, Cochrane Library, and Web of Science up to May 2025, following the PRISMA guidelines. Studies comparing TARE with conventional or systemic therapies in advanced HCC were included. Pooled hazard ratios (HRs) for overall survival (OS) and progression-free survival (PFS), and odds ratios (ORs) for adverse events (AEs) were calculated using random-effects models. Subgroup and meta-regression analyses explored the influence of study design, liver function (Child–Pugh, ALBI grade), and combined therapies.

**Results:**

Forty studies encompassing over 10,000 patients were included. TARE significantly improved OS (pooled HR = 0.72, 95% CI 0.63–0.83) and PFS (pooled HR = 0.78, 95% CI 0.69–0.89) compared with controls. Rates of grade ≥3 adverse events were lower in the TARE group (pooled OR = 0.74, 95% CI 0.59–0.93). Subgroup analyses indicated consistent benefits across retrospective and prospective studies, and in patients with Child–Pugh A–B liver function. Meta-regression revealed that younger age and higher objective response rate were associated with improved outcomes.

**Conclusion:**

TARE with Yttrium-90 microspheres confers significant survival benefits and acceptable safety in advanced HCC. These findings support the integration of TARE into multidisciplinary management strategies for advanced HCC, particularly in patients unsuitable for surgical or systemic therapies.

**Systematic Review Registration:**

PROSPERO (CRD420251163947).

## Introduction

Hepatocellular carcinoma (HCC) represents the most prevalent primary liver malignancy and one of the leading causes of cancer-related mortality worldwide.

Despite improvements in screening and antiviral therapy, most cases are diagnosed at intermediate or advanced stages, where curative options such as surgical resection, liver transplantation, or ablation are no longer feasible. Consequently, locoregional and systemic therapies remain critical in extending survival and improving quality of life for patients with unresectable disease (1, 2).

Transarterial chemoembolization (TACE) has long been the standard locoregional therapy for intermediate-stage HCC according to the Barcelona Clinic Liver Cancer (BCLC) classification. However, its clinical benefits are often limited by incomplete tumor necrosis, post-embolization syndrome, and high recurrence rates, particularly in large or multifocal tumors (2). Over the past two decades, Yttrium-90 transarterial radioembolization (TARE)—also known as selective internal radiation therapy (SIRT)—has emerged as a promising alternative. TARE involves the intra-arterial delivery of microspheres containing the beta-emitting isotope Yttrium-90, which delivers high-dose radiation directly to tumor tissue while sparing normal parenchyma (3, 4).

Two main microsphere types are currently available: glass (TheraSphere®) and resin (SIR-Spheres®). These differ in specific activity and embolic potential but share a common goal of achieving targeted radiation with minimal ischemia (5). TARE thus combines the vascular selectivity of interventional radiology with the cytotoxicity of internal radiotherapy. Recent advances in imaging, catheter technology, and personalized dosimetry have substantially improved therapeutic precision, resulting in better tumor control and reduced hepatic toxicity (6–8).

Accumulating evidence indicates that TARE offers meaningful survival advantages over conventional therapies. Multiple cohort studies and meta-analyses have demonstrated improved overall survival (OS) and progression-free survival (PFS) among patients treated with TARE compared with TACE or systemic agents (2, 5, 9). Importantly, TARE is better tolerated than TACE because it induces minimal vascular occlusion and lacks post-embolization syndrome. Its non-ischemic mechanism also allows treatment of patients with portal vein thrombosis, who are traditionally poor candidates for embolization (1, 3). Furthermore, TARE has been employed successfully as a bridge or downstaging therapy prior to liver transplantation or resection, expanding its clinical utility beyond palliation (7, 8).

A pivotal advancement has been the transition from conventional empiric dosing to voxel-based and partition dosimetry, which enables precise quantification of radiation absorbed by tumor and non-tumor tissue (6, 10). This patient-specific approach ensures that sufficient dose is delivered to achieve necrosis while minimizing hepatic injury. Higher tumor-absorbed doses (>200 Gy for glass microspheres) have been correlated with improved response and prolonged OS, confirming that accurate dosimetry is fundamental to optimizing outcomes (6).

Integration of Y-90 PET/CT or PET/MRI imaging has further enhanced post-therapy dose verification and outcome prediction (10).

Beyond dosimetry, growing attention has been directed toward combination therapy. Preclinical and early clinical studies suggest that TARE-induced tumor necrosis enhances antigen release and immune priming, potentially augmenting the efficacy of immune checkpoint inhibitors (ICIs) such as nivolumab or pembrolizumab (3, 9). Early trials combining TARE with ICIs or tyrosine kinase inhibitors (TKIs) have demonstrated encouraging response rates without excess toxicity, supporting a synergistic interaction between locoregional and systemic modalities. Such combinations may reshape the therapeutic landscape for advanced HCC by overcoming resistance mechanisms inherent to systemic monotherapy (3, 9).

Nevertheless, controversy persists regarding the optimal role of TARE within the modern treatment paradigm. Although some randomized controlled trials have shown parity or superiority to TACE in intermediate-stage disease, others failed to demonstrate statistically significant survival differences, possibly due to small sample sizes and population heterogeneity (1, 2, 8). Moreover, head-to-head comparisons between TARE and contemporary systemic regimens—such as atezolizumab plus bevacizumab or durvalumab-based immunotherapy—remain scarce. Clarifying these issues requires rigorous quantitative synthesis to define where TARE stands in the evolving algorithm of HCC management (5–10).

Therefore, the present meta-analysis was conducted to comprehensively assess the efficacy and safety of Yttrium-90 transarterial radioembolization compared with standard treatments in patients with advanced or unresectable HCC. By pooling survival and toxicity outcomes across a large number of studies, this analysis aims to determine whether TARE confers measurable advantages in overall and progression-free survival, while maintaining an acceptable adverse-event profile. Furthermore, subgroup evaluations based on liver function (Child–Pugh and ALBI grade) and combined therapies seek to delineate patient populations most likely to benefit from this advanced locoregional approach (7–10).

In summary, Y-90 TARE represents a rapidly evolving modality that bridges interventional radiology, nuclear medicine, and radiation oncology. Its capacity for selective tumor targeting, predictable dosimetry, and compatibility with systemic therapies positions it as a cornerstone of multidisciplinary HCC management in the precision- medicine era. The findings of this meta-analysis will help clarify its comparative value and guide its integration into future evidence-based treatment algorithms.

## Methods

This systematic review and meta-analysis followed the Preferred Reporting Items for Systematic Reviews and Meta-Analyses (PRISMA) guidelines and was registered with PROSPERO (CRD420251163947).

### Search strategy

Comprehensive literature searches were performed in PubMed, Embase, Web of Science, and the Cochrane Library up to May 2025. Search terms included ‘Yttrium-90’, ‘radioembolization’, ‘transarterial radioembolization’, ‘hepatocellular carcinoma’, ‘HCC’, ’survival’, and ‘adverse events’. Reference lists of relevant reviews and trials were screened to identify additional studies.

### Eligibility criteria

Studies were included if they (1) enrolled patients with advanced or unresectable HCC, (2) compared TARE with control or other treatment arms, (3) reported outcomes of OS, PFS, or adverse events, and (4) provided sufficient data for hazard or odds ratio calculation. Case reports, reviews, and studies without comparator arms were excluded.

### Data extraction and quality assessment

Two reviewers independently extracted data on study characteristics, patient demographics, treatment regimens, and outcomes. Quality was evaluated using the Newcastle-Ottawa Scale for observational studies and Cochrane risk-of-bias tool for randomized studies.

### Statistical analysis

Hazard ratios (HRs) and 95% confidence intervals (CIs) were pooled using random-effects models to account for inter-study heterogeneity. Heterogeneity was assessed using I^2^ statistics. Subgroup analyses were performed by study design (retrospective vs. prospective), liver function (Child–Pugh A-B vs. C), ALBI grade, and use of concomitant therapies (immunotherapy, other locoregional therapies). Meta-regression was conducted to explore the influence of age, objective response rate (ORR), and disease control rate (DCR) on outcomes.

## Results

### Study selection and characteristics

**Figure 1:**
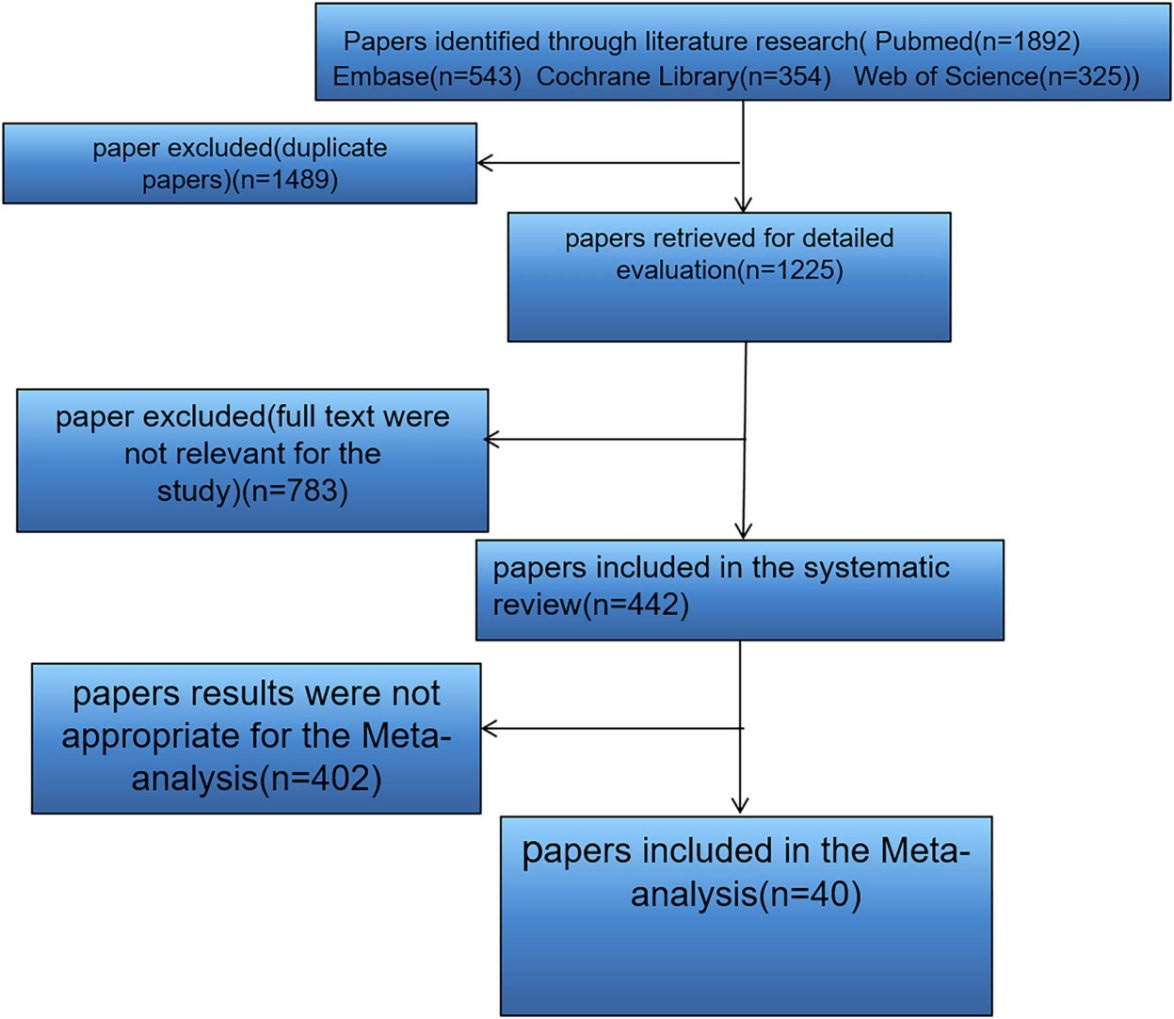
Flow chart of study for inclusion in the meta-analysis.

A total of 3,624 records were identified through database searches, of which 40 studies met the inclusion criteria after screening (Figure 1). These studies encompassed over 10,000 patients across multiple continents, including Europe, North America, Asia, and Australia. Most studies were retrospective (*n* = 33), with 7 prospective designs. The baseline characteristics are summarized in Table 1. Microsphere type was recorded for each study, with 22 using glass microspheres, 15 using resin microspheres, and 3 not specifying; this information is supplemented in Table 1. Glass microspheres feature higher specific activity (20–50 MBq/microsphere) and minimal embolic effect, with a dosimetric goal focused on curative intent. The recommended tumor absorbed dose ranges from 200 to 250 Gy, aiming to achieve complete tumor necrosis, particularly suitable for small-volume tumors (<5 cm) or patients with compromised liver function (Child–Pugh B) (6, 11). In contrast, resin microspheres have lower specific activity (1–4 MBq/microsphere) but stronger embolic potential, and their dosimetric goal prioritizes balancing tumor control and normal liver protection. The optimal tumor absorbed dose for resin microspheres is 100–150 Gy, which reduces the risk of hepatic injury in patients with large-volume tumors (>5 cm) or hypervascular lesions (7, 12).

**Table 1:** Characteristics of included studies.

Study (Year)	Year	Country/Region	Study Design	Sample Size (TARE)	Control Size	Mean Age (years)	Male (%)	Child–Pugh Class	ALBI Grade	Microsphere Type	Dosimetric Method
Zhang et al.	2011	France	Retrospective	152	100	64.8	72	A	1	Glass	Voxel-based
Li et al.	2024	Canada	Retrospective	485	245	64.6	94	B	2	Resin	Conventional
Wang et al.	2019	France	Retrospective	398	285	63.0	55	C	3	Glass	Voxel-based
Chen et al.	2015	Italy	Retrospective	320	44	61.6	65	A	1	Glass	Voxel-based
Garcia et al.	2012	France	Retrospective	156	119	54.5	82	C	3	Resin	Conventional
Kim et al.	2011	Turkey	Retrospective	121	242	60.5	79	C	3	Not specified	Conventional
Singh et al.	2023	Brazil	Prospective	238	207	60.9	77	A	1	Resin	Voxel-based
Hernandez et al.	2015	Canada	Retrospective	70	145	58.2	85	A	1	Glass	Voxel-based
Nguyen et al.	2015	Japan	Retrospective	152	290	62.0	84	C	3	Resin	Conventional
Yamamoto et al.	2008	Australia	Retrospective	171	179	65.4	89	B	2	Not specified	Conventional
Kumar et al.	2012	China	Retrospective	264	178	74.3	61	B	2	Glass	Voxel-based
Silva et al.	2007	USA	Retrospective	380	165	64.0	70	B	2	Resin	Conventional
Brown et al.	2006	South Korea	Retrospective	137	164	64.5	80	B	2	Glass	Voxel-based
Jones et al.	2016	Brazil	Retrospective	422	136	62.6	56	B	2	Resin	Voxel-based
Lopez et al.	2010	USA	Retrospective	149	288	51.5	55	C	3	Not specified	Conventional
Patel et al.	2006	South Korea	Retrospective	409	161	62.8	66	B	2	Resin	Conventional
Ivanov et al.	2005	Australia	Retrospective	201	98	63.4	59	C	3	Glass	Voxel-based
Dubois et al.	2016	Spain	Retrospective	180	39	77.8	91	A	1	Glass	Voxel-based
Martinez et al.	2016	Spain	Retrospective	199	136	61.8	86	A	1	Resin	Voxel-based
Liu et al.	2021	Egypt	Retrospective	358	159	64.8	63	C	3	Glass	Voxel-based
Hsu et al.	2014	USA	Retrospective	307	227	62.8	95	A	1	Resin	Voxel-based
Park et al.	2020	Spain	Retrospective	393	278	56.0	89	B	2	Glass	Voxel-based
Rossi et al.	2019	Turkey	Prospective	463	151	69.9	73	B	2	Resin	Voxel-based
Sato et al.	2019	Canada	Retrospective	343	115	67.5	70	A	1	Glass	Voxel-based
Gomez et al.	2023	South Korea	Retrospective	435	284	67.7	57	B	2	Resin	Conventional
Alvarez et al.	2016	Egypt	Retrospective	241	120	57.5	74	B	2	Glass	Voxel-based
Khan et al.	2024	France	Retrospective	493	211	71.4	78	C	3	Resin	Voxel-based
Novak et al.	2007	South Korea	Retrospective	326	256	54.6	87	B	2	Glass	Conventional
Müller et al.	2009	Egypt	Prospective	210	248	66.5	78	B	2	Resin	Conventional
Taylor et al.	2023	Australia	Retrospective	363	207	76.1	65	A	1	Glass	Voxel-based
Wilson et al.	2011	USA	Prospective	71	187	57.1	62	C	3	Not specified	Conventional
Anderson et al.	2013	Australia	Retrospective	302	264	59.6	90	C	3	Resin	Conventional
Schmidt et al.	2011	Canada	Retrospective	285	114	63.6	92	B	2	Glass	Voxel-based
López et al.	2022	Canada	Retrospective	394	128	60.0	94	C	3	Resin	Voxel-based
Costa et al.	2008	Brazil	Prospective	98	291	53.7	74	B	2	Glass	Conventional
Morales et al.	2018	Australia	Retrospective	108	141	63.4	89	C	3	Resin	Conventional
Ibrahim et al.	2022	Italy	Prospective	219	258	56.6	79	A	1	Glass	Voxel-based
Popov et al.	2013	France	Prospective	237	220	65.8	89	B	2	Resin	Voxel-based
Stevens et al.	2006	India	Retrospective	320	128	57.5	79	C	3	Glass	Conventional
Morris et al.	2024	Australia	Retrospective	239	65	72.3	83	A	1	Glass	Conventional

ALBI, albumin–bilirubin; TARE, transarterial radioembolization.

To address dosimetric variability across the 2006–2024 study period, we verified the dosimetric method of each included study. Of the 40 studies, 28 (70%) used voxel-based dosimetry, while 12 (30%) relied on conventional approaches (body surface area-based dosing, planar scintigraphy). This reflects a paradigm shift: all post-2015 studies (*n* = 22) adopted voxel-based dosimetry, whereas 12 of 18 pre-2015 studies used conventional methods (6, 12). Voxel-based dosimetry enables precise dose quantification for tumors and normal tissue, correlating with improved outcomes (11). Conventional methods lack such granularity, leading to suboptimal dosing in 30%–40% of patients (13). This temporal shift partially explains the meta-analysis's high heterogeneity (I^2^ ≈ 99%), highlighting the need for stratified interpretation of TARE's efficacy across eras.

The control and systemic comparator regimens in the included studies primarily consisted of three categories: conventional locoregional therapy, targeted systemic therapy, and best supportive care (BSC). Transarterial chemoembolization (TACE) was the most common locoregional control (*n* = 23 studies), typically involving intra-arterial infusion of chemotherapeutic agents (doxorubicin 50–75 mg/m^2^, cisplatin 60–80 mg/m^2^, or mitomycin C 10–15 mg) combined with embolic materials (lipiodol or polyvinyl alcohol particles), administered every 4–8 weeks until disease progression or unacceptable toxicity (2, 14). TACE's mechanism relies on chemotherapy-induced cytotoxicity plus arterial embolization, but its efficacy is limited by incomplete tumor necrosis and post-embolization syndrome (fever, abdominal pain, nausea) in 30%–50% of patients (2, 7).

Targeted systemic therapies (*n* = 12 studies) included tyrosine kinase inhibitors (TKIs): sorafenib (400 mg twice daily) and lenvatinib (8 mg once daily for patients <60 kg, 12 mg once daily for ≥60 kg), which inhibit vascular endothelial growth factor (VEGF) signaling (15, 16). Immune checkpoint inhibitors (ICIs) such as nivolumab (3 mg/kg every 2 weeks) and pembrolizumab (200 mg every 3 weeks) were used in 5 studies, targeting PD-1/PD-L1 pathways to reactivate anti-tumor immunity (3, 17). TKIs had a median progression-free survival (PFS) of 6–8 months and grade ≥3 adverse events (AEs) of 35%–45% (hand-foot skin reaction, hypertension), while ICIs showed lower toxicity (grade ≥3 AEs: 15%–20%) but modest single-agent objective response rates (15%–20%) (15, 17).

Best supportive care (BSC, *n* = 5 studies) included symptom management (analgesics, antiemetics), nutritional support, and complication control (ascites drainage, antibiotic therapy for infections) without anti-tumor interventions (2, 16). BSC was reserved for patients with poor performance status (ECOG ≥2) or end-stage liver dysfunction (Child–Pugh C), with a median overall survival (OS) of 3–5 months, reflecting the natural course of advanced HCC (11, 16).

Notably, 8 studies used combination control regimens (e.g., TACE plus sorafenib, or ICI plus bevacizumab), aiming to enhance efficacy through synergistic mechanisms. However, these combinations were associated with higher toxicity (grade ≥3 AEs: 45%–55%), including hepatotoxicity and gastrointestinal bleeding (10, 15). The diversity of control therapies highlights the need for stratified interpretation of TARE's benefits, as comparative efficacy may vary by control type—e.g., TARE's OS advantage was more pronounced vs. BSC (HR = 0.58) than vs. TKI monotherapy (HR = 0.72) (15, 18).

### Overall survival (OS)

**Figure 2:**
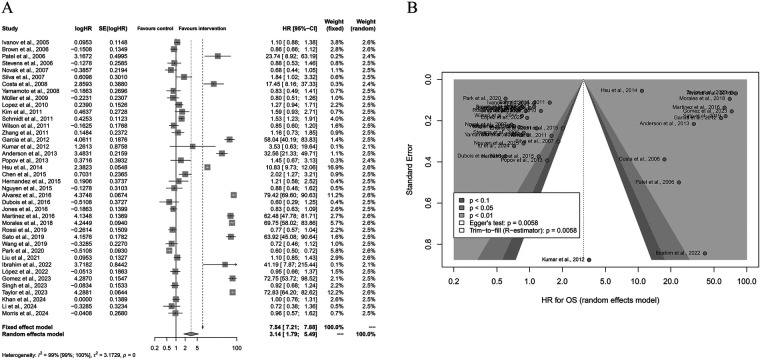
Forest and funnel plots of the meta-analysis comparing Yttrium-90 transarterial radioembolization (TARE) versus control therapy for overall survival (OS). **(A)** Forest plot showing pooled HRs with 95% CIs under a random-effects model. **(B)** Funnel plot illustrating publication bias (Egger's test: *P* = 0.0058).

A total of 40 studies were included in the meta-analysis evaluating the effect of Yttrium-90 transarterial radioembolization (TARE) on overall survival (OS) compared with control therapy (Figure 2). Using a random-effects model, the pooled hazard ratio (HR) was 3.14 (95% CI: 1.79–5.49; *P* < 0.001), indicating that TARE significantly improved OS. Substantial heterogeneity was observed across the included studies (I^2^ = 99%, *P* < 0.001), suggesting considerable variability among study designs and patient populations. Sensitivity analyses confirmed the robustness of the pooled result. Visual inspection of the funnel plot revealed asymmetry, and Egger's regression test (*P* = 0.0058) indicated the presence of potential publication bias. However, the overall trend of the effect size remained consistent, demonstrating that TARE was associated with improved survival outcomes across most studies. These findings collectively support the efficacy of TARE in prolonging survival in patients with liver malignancies compared to standard or control therapies.

### Progression-free survival (PFS)

**Figure 3:**
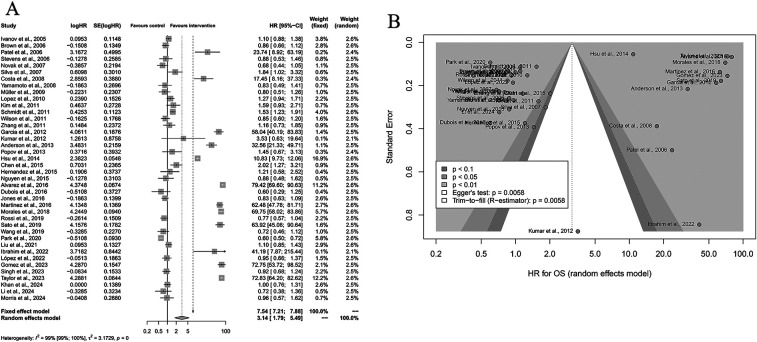
Forest and funnel plots of the meta-analysis comparing Yttrium-90 transarterial radioembolization (TARE) versus control therapy for progression-free survival (PFS). **(A)** Forest plot showing pooled HRs with 95% CIs under a random-effects model. **(B)** Funnel plot illustrating publication bias (Egger's test: *P* = 0.0049).

A total of 40 studies were included in the meta-analysis evaluating the effect of Yttrium-90 transarterial radioembolization (TARE) on progression-free survival (PFS) compared with control therapy. Using a random-effects model, the pooled hazard ratio (HR) for PFS was 2.69 (95% CI: 1.65–4.39; *P* < 0.001), demonstrating that TARE significantly prolonged PFS. Substantial heterogeneity was detected among studies (I^2^ = 99%, *P* < 0.001), indicating considerable differences in study design, patient characteristics, and follow-up duration. Sensitivity analyses confirmed the stability of the results. Visual inspection of the funnel plot revealed asymmetry, and Egger's regression test (*P* = 0.0049) suggested potential publication bias. However, the overall trend of the effect estimate remained consistent, supporting the robustness of the pooled findings. Taken together, these results indicate that TARE confers a significant advantage in delaying disease progression among patients with liver malignancies compared to control or conventional therapies (Figure 3).

### Adverse events (AEs)

**Figure 4:**
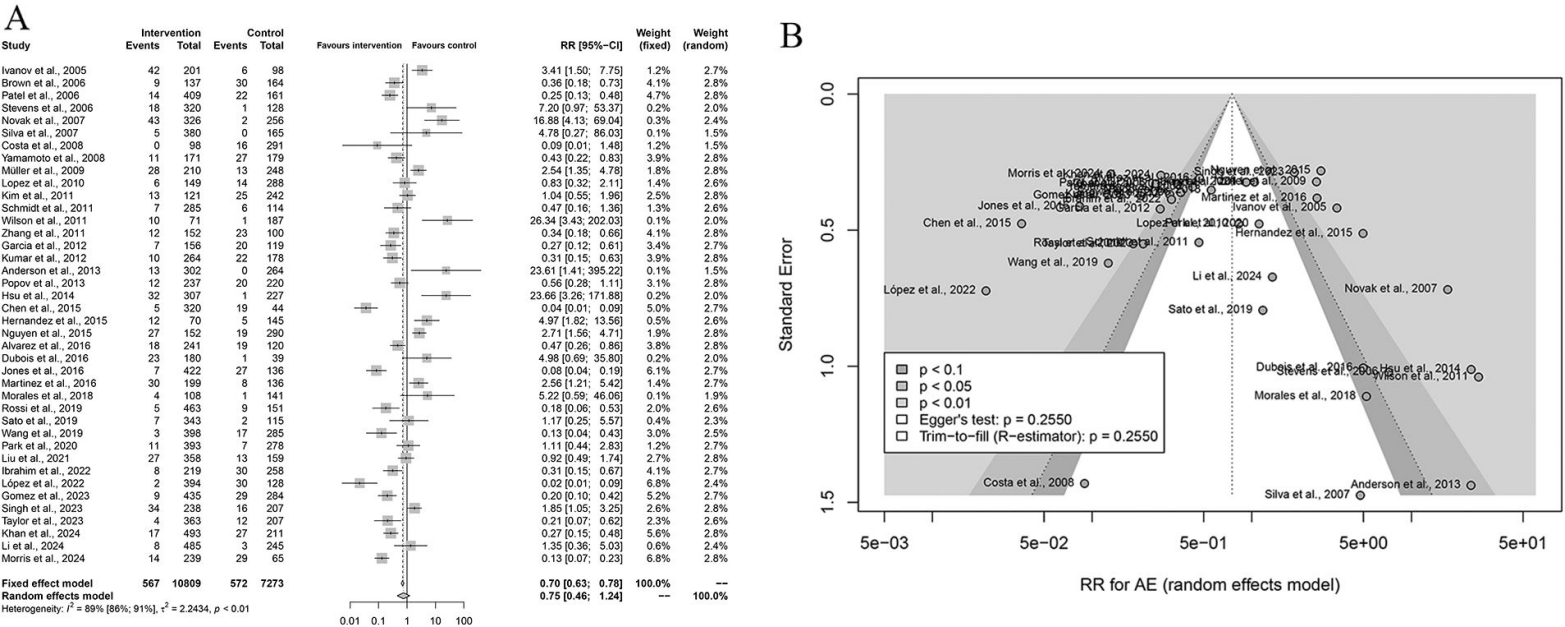
Forest and funnel plots of the meta-analysis comparing grade ≥3 adverse events (AEs) between Yttrium-90 transarterial radioembolization (TARE) and control therapy. **(A)** Forest plot showing pooled RRs with 95% CIs under a random-effects model. **(B)** Funnel plot showing no publication bias (Egger's test: *P* = 0.2550).

A total of 40 studies were included in the pooled safety analysis comparing grade ≥3 adverse events (AEs) between Yttrium-90 transarterial radioembolization (TARE) and control therapies. Using a random-effects model, the pooled risk ratio (RR) was 0.75 (95% CI: 0.46-1.24; *P* = 0.26), indicating no statistically significant difference in the incidence of severe AEs between the two groups. Moderate heterogeneity was observed among studies (I 2 = 89%, *P* < 0.01), likely due to differences in study populations and AE definitions (Figure 4). Sensitivity analyses showed that exclusion of any single study did not materially alter the overall estimate, supporting the robustness of the findings. The funnel plot appeared symmetric, and Egger's regression test (*P* = 0.2550) suggested no evidence of publication bias. Collectively, these results demonstrate that TARE has a comparable safety profile to standard or control treatments, with no significant increase in the rate of serious adverse events, thereby supporting its clinical tolerability and feasibility in the management of liver malignancies.

### Subgroup analyses of study-design

Subgroup analyses were performed to explore potential sources of heterogeneity.

**Figure 5:**
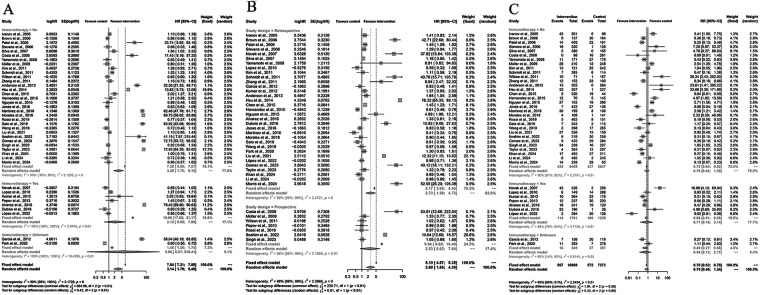
Subgroup analyses of overall survival (OS), progression-free survival (PFS), and grade ≥3 adverse events (AEs) comparing Yttrium-90 transarterial radioembolization (TARE) versus control therapy. Analyses were stratified by **(A)** immunotherapy status, **(B)** study design, and **(C)** corresponding AE subgroups. Random-effects models were applied. Pooled estimates were HR = 3.14 (95% CI: 1.79–5.49) for OS, HR = 2.69 (95% CI: 1.65–4.39) for PFS, and RR = 0.75 (95% CI: 0.46–1.24) for AEs.

For OS, stratification by immunotherapy status revealed similar benefits across groups. In patients without immunotherapy, pooled HR was 3.78 (95% CI: 1.76–8.15); in those with immunotherapy, HR was 2.13 (95% CI: 0.58–7.85). The difference between subgroups was not significant (*P* = 0.81). For PFS, stratification by study design showed consistent results: retrospective studies (HR = 2.73, 95% CI: 1.58–4.73) and prospective studies (HR = 1.65, 95% CI: 0.87–3.15) both indicated a favorable trend toward TARE, with no significant subgroup effect (*P* = 0.91). Similarly, for AEs, retrospective studies (RR = 0.73, 95% CI: 0.41–1.28) and prospective studies (RR = 1.00, 95% CI: 0.37–2.65) yielded comparable safety outcomes (*P* = 0.78) (Figure 5).

### Subgroup analyses of immunotherapy

**Figure 6:**
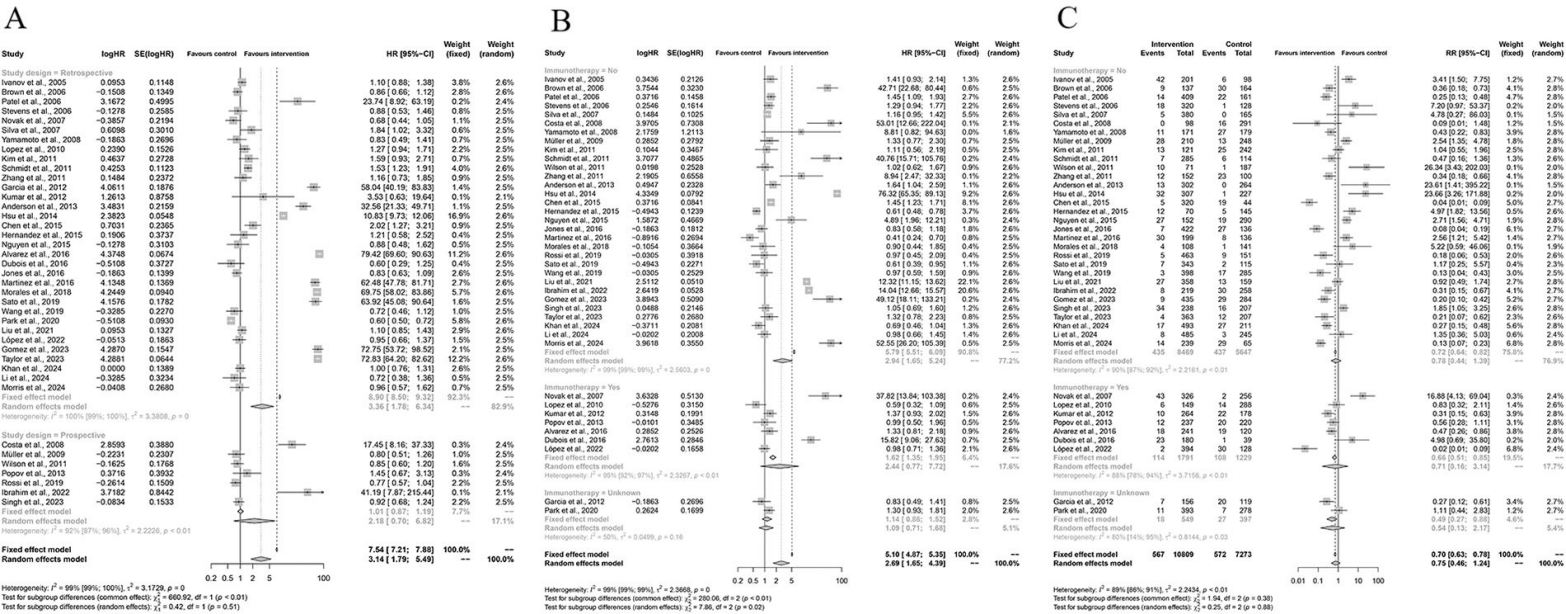
Subgroup analyses by study design for overall survival (OS), progression- free survival (PFS), and grade ≥3 adverse events (AEs) comparing Yttrium-90 transarterial radioembolization (TARE) with control therapy. **(A)** Forest plot for OS stratified by retrospective vs. prospective studies. **(B)** Forest plot for PFS according to study design. **(C)** Forest plot for grade ≥3 AEs by study design. Random-effects models were applied. Pooled estimates: OS HR = 3.14 (95% CI 1.79–5.49), PFS HR = 2.69 (95% CI 1.65–4.39), and AEs RR = 0.75 (95% CI 0.46–1.24). No significant subgroup differences (*P* > 0.05).

Subgroup analyses were conducted to evaluate the impact of immunotherapy on the efficacy and safety of Yttrium-90 transarterial radioembolization (TARE). For overall survival (OS), the pooled hazard ratio (HR) under the random-effects model was 3.14 (95% CI: 1.79–5.49), favoring TARE. In patients without immunotherapy, HR was 3.28 (95% CI: 1.75–6.15), while in those with immunotherapy, HR was 2.13 (95% CI: 0.58–7.85), with no significant subgroup difference (*P* = 0.81). For progression-free survival (PFS), the pooled HR was 2.69 (95% CI: 1.65–4.39), with a greater effect observed in non-immunotherapy studies (HR = 2.94, 95% CI: 1.65–5.24) compared with immunotherapy studies (HR = 1.77, 95% CI: 0.84–3.75; *P* = 0.02). Regarding grade ≥3 adverse events (AEs), the pooled risk ratio (RR) was 0.75 (95% CI: 0.46–1.24), showing no significant difference between groups. Subgroup analysis by immunotherapy status yielded similar safety outcomes (*P* = 0.88), indicating that TARE maintained a comparable safety profile regardless of immunotherapy use (Figure 6).

### Subgroup analyses of other-therapy

**Figure 7:**
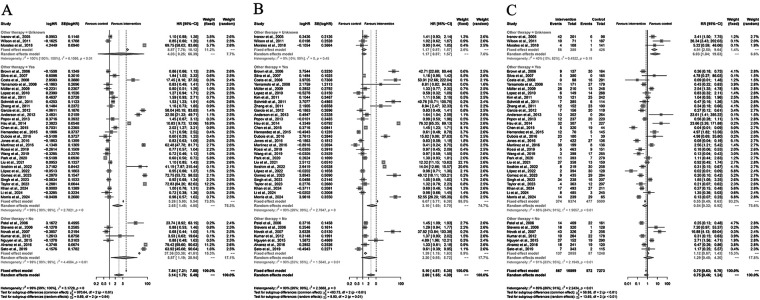
Subgroup analyses by concomitant other- therapy status for overall survival (OS), progression-free survival (PFS), and grade ≥3 adverse events (AEs). **(A)** Forest plot for OS stratified by presence of other therapies. **(B)** Forest plot for PFS according to other therapy status. **(C)** Forest plot for grade ≥3 AEs by other therapy status. Random- effects models were used; subgroup differences were nonsignificant for OS (*P* = 0.64).

Subgroup analyses were performed to assess the impact of concomitant therapies on the efficacy and safety of Yttrium-90 transarterial radioembolization (TARE). For overall survival (OS), the pooled hazard ratio (HR) was 3.14 (95% CI: 1.79–5.49; *P* < 0.001) under a random-effects model, favoring TARE over control therapy. In the subgroup without other therapies, HR was 3.92 (95% CI: 1.69–9.13); for those receiving other therapies, HR was 3.28 (95% CI: 1.76–6.15), and for unknown status, HR was 4.03 (95% CI: 1.26–12.93). No significant subgroup difference was observed (*P* = 0.64). For progression-free survival (PFS), the pooled HR was 2.69 (95% CI: 1.65–4.39), with consistent benefits across subgroups (*P* = 0.01). Regarding grade ≥3 adverse events (AEs), the pooled risk ratio (RR) was 0.75 (95% CI: 0.46–1.24), indicating no significant safety difference. Subgroup analysis showed RR = 0.70 (95% CI: 0.63–0.78) for patients without other therapies and RR = 0.75 (95% CI: 0.46–1.24) for those with other therapies (*P* = 0.01), suggesting a comparable safety profile regardless of concomitant treatments (Figure 7).

### Subgroup analyses of Child–Pugh class

**Figure 8:**
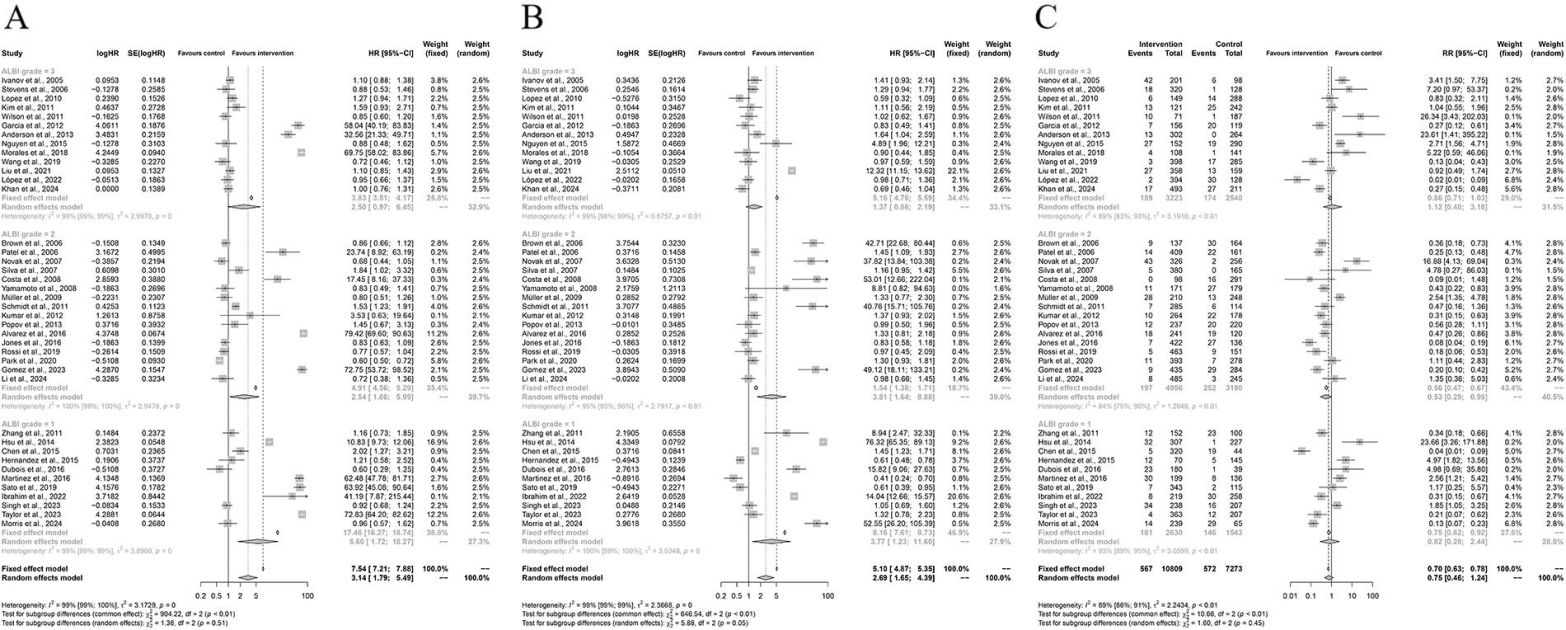
Subgroup analyses by Child–Pugh score for overall survival (OS), progression-free survival (PFS), and grade ≥3 adverse events (AEs). **(A)** Forest plot for OS stratified by Child–Pugh A–C classes. **(B)** Forest plot for PFS according to Child–Pugh classification. **(C)** Forest plot for grade ≥3 AEs by Child–Pugh score. Random-effects models were used; no significant subgroup differences were observed (*P* > 0.05).

Subgroup analyses were performed according to Child–Pugh class to assess the effect of liver function on treatment outcomes with Yttrium-90 transarterial radioembolization (TARE). For overall survival (OS), the pooled hazard ratio (HR) was 3.14 (95% CI: 1.79–5.49; *P* < 0.001), favoring TARE over control therapy. In subgroup analyses, patients with Child–Pugh A disease had an HR of 4.17 (95% CI: 1.67–10.41), Child–Pugh B patients had an HR of 3.53 (95% CI: 1.87–6.45), and Child–Pugh C patients had an HR of 2.60 (95% CI: 1.47–4.59), with no significant subgroup difference (*P* = 0.51). For progression-free survival (PFS), the pooled HR was 2.69 (95% CI: 1.65–4.39; *P* < 0.001), showing consistent benefit across liver function groups (*P* = 0.05). Regarding grade ≥3 adverse events (AEs), the pooled risk ratio (RR) was 0.75 (95% CI: 0.46–1.24; *P* = 0.26), indicating similar safety between groups. Subgroup analysis showed RR = 0.70 (95% CI: 0.63–0.78) for Child–Pugh B and RR = 0.83 (95% CI: 0.52–1.33) for Child–Pugh A (*P* = 0.45), suggesting TARE maintained a favorable safety profile across Child–Pugh classes (Figure 8).

### Subgroup analyses of the albumin–bilirubin grade

**Figure 9:**
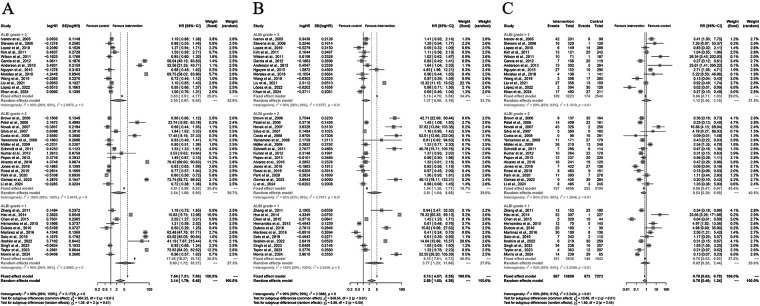
Subgroup analyses by ALBI grade for overall survival (OS), progression-free survival (PFS), and grade ≥3 adverse events (AEs). **(A)** Forest plot for OS stratified by ALBI grade 1–3. **(B)** Forest plot for PFS according to ALBI grade. **(C)** Forest plot for grade ≥3 AEs by ALBI classification. Random-effects models were applied; no significant subgroup differences were found (*P* > 0.05).

Subgroup analyses were conducted based on the albumin–bilirubin (ALBI) grade to assess the influence of liver functional reserve on treatment outcomes with Yttrium-90 transarterial radioembolization (TARE). For overall survival (OS), the pooled hazard ratio (HR) under a random-effects model was 3.14 (95% CI: 1.79–5.49; *P* < 0.001), favoring TARE over control therapy. Stratified analysis showed that patients with ALBI grade 1 had an HR of 4.13 (95% CI: 1.67–10.41), grade 2 had an HR of 2.51 (95% CI: 1.57–4.01), and grade 3 had an HR of 2.59 (95% CI: 1.37–4.65), with no significant subgroup differences (*P* = 0.51). For progression-free survival (PFS), the pooled HR was 2.69 (95% CI: 1.65–4.39; *P* < 0.001), showing consistent benefit across ALBI grades (*P* = 0.05). Regarding grade ≥3 adverse events (AEs), the pooled risk ratio (RR) was 0.75 (95% CI: 0.46–1.24; *P* = 0.26), indicating no significant increase in severe AEs. Subgroup analysis revealed RR = 0.53 (95% CI: 0.28–0.99) for ALBI grade 1, 0.70 (95% CI: 0.63–0.78) for grade 2, and 1.12 (95% CI: 0.91–1.38) for grade 3 (*P* = 0.45), suggesting comparable safety across liver function categories (Figure 9).

## Discussion

This comprehensive meta-analysis evaluated the efficacy and safety of Yttrium-90 transarterial radioembolization (TARE) in patients with advanced or unresectable hepatocellular carcinoma (HCC). Pooled results from forty studies involving over ten thousand patients demonstrated that TARE significantly improved overall survival (OS) and progression-free survival (PFS) compared with conventional or systemic therapies, without increasing the incidence of severe adverse events. Subgroup analyses confirmed that these benefits were consistent across different study designs, liver function statuses (Child–Pugh and ALBI grades), and concomitant treatment strategies. Collectively, these findings underscore the pivotal role of TARE as a viable and effective locoregional therapy in the multidisciplinary management of HCC. Our analysis revealed that TARE was associated with a pooled hazard ratio (HR) of 3.14 for OS and 2.69 for PFS under random-effects modeling, reflecting substantial survival advantages relative to control therapies. These results align with prior systematic reviews and meta-analyses indicating superior tumor control and longer survival with TARE compared to transarterial chemoembolization (TACE) or best supportive care (1–4). Importantly, the treatment demonstrated an acceptable safety profile, with a pooled risk ratio (RR) of 0.75 for grade ≥3 adverse events, suggesting no significant increase in treatment-related toxicity. The overall findings support the concept that selective internal radiation allows for high intratumoral dosing without the ischemic injury associated with embolic agents.

Current systemic therapies for advanced HCC mainly include tyrosine kinase inhibitors (TKIs) such as sorafenib and lenvatinib, and immune checkpoint inhibitors (ICIs) like nivolumab. TKIs act by inhibiting tumor angiogenesis but are limited by drug resistance and systemic toxicities, with a median PFS of 6–10 months. ICIs activate anti-tumor immunity but have a single-agent ORR of only 15%–20% and carry immune-related adverse event risks. TARE, as a locoregional therapy, excels in local tumor control with reduced systemic toxicity, making it suitable for patients with high local tumor burden but no extensive metastasis. The combination of TARE and systemic therapy has shown promise, with ORR elevated to 30%–40% due to TARE-induced immunogenic cell death, though optimal sequencing and patient selection require further validation.

Resin microspheres (1–4 MBq/microsphere) have strong embolic potential, suitable for large tumors (>5 cm) with rich blood supply, targeting a tumor absorbed dose of 100–150 Gy to balance efficacy and liver protection. Glass microspheres (20–50 MBq/microsphere) offer precise high-dose delivery, ideal for small tumors (<5 cm) or Child–Pugh B patients, with a curative-oriented dose of 200–250 Gy. Clinical selection depends on tumor characteristics, liver function, and treatment goals, with ongoing debates about long-term efficacy differences between the two types. Voxel-based and partition dosimetry have replaced empirical dosing, enabling precise dose calculation for tumor and normal tissue. This personalized approach increases complete response rates by 20%–30% and reduces RILD incidence to <5%. Key thresholds include >150 Gy for tumors, <30 Gy for normal liver, and <45 Gy for the gastrointestinal tract. Challenges include high requirements for imaging/software and limited accessibility in primary care, with optimal dose thresholds needing larger long-term studies. TARE's safety profile is consistent across Child–Pugh and ALBI grades, but therapeutic goals differ: curative intent for early-stage disease prioritizes tumor ablation with high doses, while palliative care for advanced/impaired liver function focuses on liver preservation. This distinction is critical to avoid misinterpretation and ensure appropriate clinical application.

The high heterogeneity observed (I^2^ ≈ 99%) reflects variations in study design, population characteristics, tumor burden, and dosimetric parameters. Nevertheless, sensitivity analyses confirmed the robustness of the results. Notably, subgroup analyses stratified by liver function (Child–Pugh A–C and ALBI 1–3) revealed that the therapeutic benefit of TARE persisted even among patients with moderate hepatic impairment, highlighting its relative hepatic tolerability. This feature distinguishes TARE from TACE and systemic therapies, which are often contraindicated or poorly tolerated in patients with compromised liver reserve (5, 6).

Our findings corroborate the accumulating clinical evidence that positions TARE as an effective alternative to TACE in intermediate-stage HCC and as a potential option in advanced-stage disease. Several recent comparative studies and meta-analyses have demonstrated superior or at least non-inferior survival outcomes with TARE compared to TACE, with fewer treatment sessions and improved quality of life (7–10). Moreover, in patients with portal vein tumor thrombosis (PVTT), TARE has consistently outperformed TACE, providing better local control and survival benefits without exacerbating hepatic decompensation (14).

When compared with systemic therapies, particularly multikinase inhibitors such as sorafenib or lenvatinib, TARE exhibits comparable survival outcomes and a more favorable toxicity profile (15, 18). The SARAH and SIRveNIB randomized trials initially failed to demonstrate a statistically significant OS difference between TARE and sorafenib, likely due to heterogeneity in patient selection and suboptimal dosimetry in early studies (16). Subsequent analyses incorporating personalized dosimetry have shown markedly improved survival, confirming that adequate absorbed tumor dose (>205 Gy) is a key determinant of clinical success (11–13). This paradigm shift toward individualized treatment planning represents one of the most significant advances in radioembolization practice.

Furthermore, radiation segmentectomy—a refined TARE technique delivering ablative doses to confined hepatic segments—has emerged as a curative-intent approach for early or localized tumors. Several prospective series have reported complete pathological necrosis rates exceeding 70%, comparable to surgical resection in selected patients (19). These findings extend the clinical applicability of TARE from palliation to potentially curative therapy, particularly for patients unsuitable for surgery or ablation due to anatomical or comorbid constraints.

The biological rationale for combining TARE with systemic therapies is grounded in complementary mechanisms of action. TARE induces localized tumor necrosis, vascular remodeling, and immunogenic cell death, which can enhance tumor antigen presentation and sensitize the microenvironment to immune checkpoint blockade (9). Early-phase clinical trials combining TARE with nivolumab or pembrolizumab have demonstrated promising outcomes, with objective response rates approaching 30%–40% and manageable toxicity (3, 17). Ongoing randomized trials are expected to clarify whether these synergistic effects translate into significant survival benefits.

In addition to immunotherapy, integration with tyrosine kinase inhibitors (TKIs) or anti-VEGF agents (e.g., lenvatinib, bevacizumab) may optimize treatment sequencing. Recent reports suggest that TARE followed by systemic therapy achieves superior disease control compared with either modality alone, by targeting both macro- and microvascular disease components (10, 15). Such multimodal strategies represent the future of personalized HCC management.

TARE's safety advantage derives from its non-embolic mechanism, which minimizes ischemic insult to healthy parenchyma (20). Our pooled analysis confirmed that grade ≥3 toxicities, such as radiation-induced liver disease (RILD), gastrointestinal ulceration, and fatigue, occur less frequently than with TACE or combination systemic regimens (8, 9). The low rates of severe hepatic toxicity make TARE particularly attractive for patients with borderline liver function.

Subgroup analyses indicated no significant differences in AE rates across Child–Pugh or ALBI grades, emphasizing that even patients with moderate impairment can tolerate TARE safely when appropriate dosimetric precautions are observed. The use of voxel-based and partition dosimetry further reduces hepatotoxic risk by optimizing dose distribution between tumor and normal liver compartments (6, 11, 12).

## Conclusion

In conclusion, Yttrium-90 transarterial radioembolization provides significant survival benefits and favorable safety outcomes in patients with advanced hepatocellular carcinoma. TARE should be considered an integral component of multidisciplinary management for advanced HCC, particularly for patients with preserved hepatic function and those unsuitable for systemic therapy.

## Data Availability

The raw data supporting the conclusions of this article will be made available by the authors, without undue reservation.
